# 302. Investigating NAI Titer as a Neutralizing IAV Correlate of Protection

**DOI:** 10.1093/ofid/ofad500.374

**Published:** 2023-11-27

**Authors:** Kalee E Rumfelt, Adam S Lauring, Emily T Martin, Arnold Monto, Joshua Petrie

**Affiliations:** University of Michigan - School of Public Health, Dexter, Michigan; University of Michigan, Ann Arbor, MI; University of Michigan, Ann Arbor, MI; University of Michigan, Ann Arbor, MI; Marshfield Clinic Research Institute, Marshfield, Wisconsin

## Abstract

**Background:**

Influenza vaccines are updated each year to maximize protection against circulating strains. This process relies on Correlates of Protection (CoPs) that compare an assay’s measure of an immune response with the level of protection. Three common CoPs for Influenza A Virus (IAV) are Hemagglutinin (HA) Inhibition (HAI), Neuraminidase (NA) Inhibition (NAI), and microneutralization (MN) titers. These capture different aspects of the immune response, and the degree to which NA antibodies provide protection is not well understood. Determining how these assays are related has implications for understanding the contribution of multiple antibody-based mechanisms of protection against infection.

**Methods:**

Pre-/ post-season sera were collected from individuals enrolled in the HIVE study for the 2016-17 and 2017-18 seasons. HAI and NAI assays were performed on 216 samples from 139 individuals for A/Hong Kong/4801/2014 and A/Singapore/INFIM-16-0019/2016. MN assays were performed on 179 samples from 81 individuals using A/Hong Kong. Linear regression was used to assess the association between the NAI and MN titers, controlling for HAI titers. Descriptive statistics were used to determine the level of antibody cross-reactivity of the subsequently-circulating A/Singapore NA, with a 4-fold difference in titer indicating discordance.

**Results:**

After adjusting for age, the regression model produced beta estimates of 0.16 for HAI and 0.54 for NAI titers, but neither were significant (HAI p= 0.09 NAI p= 0.08). The percentage of individuals with NAI titers that were discordant for A/Hong Kong was 14.8% in 2016-17 and 20.8% in 2017-18. No individuals had A/Singapore titers that were discordant compared to A/Hong Kong titers.

**Conclusion:**

Although not significant, we found NAI assay titers were more strongly associated with LMN titers, suggesting that NA antibodies provide some degree of neutralizing activity. A small proportion of individuals had discordant NAI results, which indicate limited cross-reactivity across strains. This raises questions regarding differences in antibody production toward differing NAs. The findings of this study suggest that NA antibodies are capable of neutralizing IAV and this activity differs across strains and individuals.

**Disclosures:**

**Adam S. Lauring, MD, PhD**, Roche: Advisor/Consultant|Sanofi: Advisor/Consultant **Emily T. Martin, PhD, MPH**, Merck: Grant/Research Support **Arnold Monto, MD**, Roche: Advisor/Consultant|Roche: Honoraria **Joshua Petrie, PhD**, CSL Seqirus: Grant/Research Support

